# Rapid screening for antigenic characterization of GII.17 norovirus strains with variations in capsid gene

**DOI:** 10.1186/s13099-022-00504-1

**Published:** 2022-07-25

**Authors:** Yingyin Liao, Liang Xue, Junshan Gao, Yueting Zuo, Yanhui Liang, Yueting Jiang, Weicheng Cai, Jiale Yang, Jumei Zhang, Yu Ding, Moutong Chen, Aiwu Wu, Xiaoxia Kou, Qingping Wu

**Affiliations:** 1 Guangzhou Key Laboratory for Clinical Rapid Diagnosis and Early Warning of Infectious Diseases, KingMed School of Laboratory Medicine, Guangzhou Medical University, Guangzhou, China; 2 Guangdong Provincial Key Laboratory of Microbial Safety and Health, State Key Laboratory of Applied Microbiology Southern China, Guangdong Institute of Microbiology, Guangdong Academy of Sciences, Guangzhou, China; 3 Department of Laboratory Medicine, First Affiliated Hospital of Guangzhou Medical University, Guangzhou, 510120 China

**Keywords:** Evolution, Norovirus, Cell-free protein synthesis system, Immune evasion

## Abstract

The emergence of the novel GII.17 Kawasaki 2014 norovirus variant raising the interest of the public, has replaced GII.4 as the predominant cause of noroviruses outbreaks in East Asia during 2014–2015. Antigenic variation of the capsid protein is considered as one of the key mechanisms of norovirus evolution. In this study, we screened a panel of GII.17 mutants. First, we produced norovirus P proteins using cell-free protein synthesis (CFPS) system, comparing the results to pure proteins expressed in a cell-based system. Next, we determined the binding capability of specific monoclonal antibody (mAb) 2D11 using a unique set of wild-type GII.17 strains. Results of the EIA involving a panel of mutant cell-free proteins indicated that Q298 was the key residue within loop 1. These data highlighted the essential residues in the linear antibody binding characteristics of novel GII.17. Furthermore, it supported the CFPS as a promising tool for rapidly screening mutants via the scalable expression of norovirus P proteins.

**Supplementary Information:**

The online version contains supplementary material available at 10.1186/s13099-022-00504-1.

## Introduction

Worldwide, human noroviruses are responsible for 16% of the sporadic cases and outbreaks of acute gastroenteritis [1]. Although the norovirus illnesses are self-limiting in healthy individuals, the risk they pose to young children, elderly people, and immunocompromised patients cannot be ignored [2, 3]. Annually, approximately 200,000 children deaths resulted by noroviruses and bring a huge social burden of 60 billion dollars [4]. A low infectious dose and easily transmissibility make challenging to control the noroviruses burden [5]. However, efforts to develop noroviruses vaccines were hindered due to multiple factors [6, 7].

Noroviruses consist of at least 31 genotypes in the two major noroviruses genogroups, GI and GII. For the past two decades, the GII.4 genotype has been dominant worldwide, leading ~ 75% of noroviruses infections [8, 9]. However, the emergence of novel GII.17 catching publics’ attention, has outcompeted GII.4 as the predominant cause of noroviruses outbreaks in several countries in late 2014 [10]. The first GII.17 strain reported in GenBank was Hu/GII.17/C142/1978/GUF (GII.17.1978). Then the strain stayed “antigenically static” until sufficient point insertions and deletions in its capsid gene to change the pattern [11, 12]. Residues substitutions at and around binding sites of histo-blood group antigens (HBGAs) and antigenic features may drive the emergence of a new pandemic variant emergence or immune evasion. Although the novel GII.17 strains seem to infect new target populations continually with altered ligand binding properties and antigenicity [13, 14], these sites have not been investigated. Findings of extensive antigenic diversity between various noroviruses strains and the interrelationships between herd protective immunity and the virus antigenic heterogeneity are warranted, which assist in developing noroviruses vaccine and revealing the evolutionary mechanisms of the virus.

Researches on virus-like particles (VLPs) which mirror in morphology and antigenicity to natural virions, are the reliable and robust approaches to shed light on the evolutionary mechanisms of noroviruses and control the burden of infections. P domain resides the outmost of the virion, which replicates many details of VLP, including antibody binding sites [15]. Although the number of P protein and VLP applications has been rapidly grown and advance the vaccine development, the production procedures are time-consuming. Evolutionary mechanism might reach a further step if a rapid screening method is applied.

In the absence of culture methods and a small animal model, mutagenesis analysis is broadly used to explore norovirus’s antigenic features. As mentioned above, P proteins expressed by *E. coli* system and VLP expressed by insect cells are time-consuming. To further explore GII.17 antigenic variation, we applied the cell-free protein synthesis (CFPS) system, an alternative to express panels P domain proteins with mutations; this system is widely known for its high productivity and is increasingly used in research. As the platform is an “open” system, the additional components, such as essential energy and modified amino acids can be supplemented conveniently. The most commonly used system, the *E. coli* S30 extract-based CFPS system, offers the highest protein yield [16] and is more suitable for producing mutant strains with which binding assays using monoclonal antibodies (mAbs) [17, 18].

Herein, we aimed to provide valuable antigenic features of the 2014–2015 GII.17 pandemic using GII.17 P proteins and *E. coli* S30 extract-based CFPS system. Combined with bioinformatics analyses, functional regions of GII.17 capsid gene were predicted and a panel of mutant GII.17 variants with chimeric regions were generated. An enzyme immunoassay (EIA) was then used to define the potential new antigenic features of GII.17 with mouse selected monoclonal antibodies (mAbs). Finally, the function of region screening by the CFPS system was confirmed by the cell-based system.

## Materials and methods

### Data collection and preparation for bioinformatics analysis

All full-length nucleotide sequences of the GII.17 *VP1* region (> 1560 bp) were obtained from GenBank. The sequences were downloaded with the information of the accession number, collection date and geographical region. Accession numbers of sequences for phylogenetic tree construction were listed in Additional File 1. To minimize the number of the sequences with high similarity, CD-HIT software [19] was used to remove redundant sequences with a threshold of 99% similarity. Multiple sequences of the constructed dataset were aligned using Multiple Alignment in Fast Fourier Transform (MAFFT) software [20].

### Evolutionary analysis

A best-fit nucleotide substitution model was determined using Akaike information criterion (AIC) as implemented in jModelTest [21], which was used to reconstruct a maximum-likelihood (ML) tree with a bootstrap analysis of 1000 replicates using PhyML version 3.0 [22]. Phylogenetic trees were analyzed using Bayesian inference via the Markov Chain Monte Carlo (MCMC) method in the BEAST 1.10.4 software [23]. Best substitution models (GTR + G) were determined for the selected sequences by comparison of the Bayesian Information Criterion (BIC) values using jModelTest. Molecular clock models calculated the nucleotide substitution rates of norovirus GII.17, as well as the time to the most recent common ancestor (TMRCA). The MCMC runs were performed with chain lengths of 40,000,000 steps, sampling every 4000 steps, for the selected sequences. Convergence of parameters were analyzed in the Tracer version 1.6 (http://tree.bio.ed.ac.uk/software/tracer/) and effective sample sizes (ESS) values of 200 or more were accepted. 95% highest probability density (HPD) values were used to estimate statistical uncertainty in parameter values. Files were combined in LogCombiner v1.10.4, and the maximum clade credibility trees were generated by discarding the first 10% of trees using TreeAnnotator v1.10.4. The tree was visualized in the FigTree 1.4.2.

### Plasmid construction and CFPS reactions

A new emerging GII.17 was obtained from stool sample in a continuous norovirus surveillance study in Guangzhou, China of 2014–2015 [24]. Norovirus was detected in stool samples by targeting region A and C. Viral RNA was extracted from clarified supernatant using QIAamp Viral RNA MiniKit (QIAgen, Hilden, Germany). Whole-genome sequences of GII.17 were obtained using a newly established “4 + 1 + 1” PCR strategy [25]. Other GII.17 capsid genes were synthesized chemically through Generay following codon optimization. Then we cloned the GII.17 P domain construct (amino acids 222–540) of GII.17 into the pET-22b ( +) expression vector using Nde I and Xho I restriction sites.

Proteins were expressed from the plasmid using the *E. coli* S30 Extract System (Promega, USA), incubated at 37℃ for 1 h with shaking at 1200 rpm in a 1.5 mL Eppendorf tube. A standard reaction comprised 18 µL of *E. coli* S30 lysate, 20 µL of reaction mix with amino acids, and 1 µg of the plasmid template harboring the target gene, made to 50 µL with water.

### Protein aggregation analysis and pure P proteins preparation

To quantify protein yield, the cell-free reaction mixture was used to perform an EIA with anti-GII.17 mAbs (mAb 2A11 and mAb 2D11, Genecreat, China). The stool sample of positive norovirus cases were subjected to RT-PCR to obtain the ORF1 region. We then cloned capsid gene of GII.17 and expressed proteins. Mouse were immunized with GII.17 antigen and splenocytes were isolated from the mouse, then fused with myeloma. We generated a dose–response curve by using various concentrations of pure GII.17-L343/P protein binding to mAb. Pure proteins were diluted using “empty” cell-free reaction mixture to create a similar procedure to that of other cell-free proteins. Pure proteins were expressed using a pGEX-4T-1/GII.17 clade D P recombinant in *E. coli* strain BL21 and purified using affinity chromatography. The GST tag was removed from target proteins using Thrombin (GE Healthcare Life Sciences) at 22℃ for 20 h. Subsequently, the expression level of cell-free proteins was quantified using the “standard curve”.

### Enzyme immunoassay

The binding assay was carried out using recombinant P domain proteins (wild-type and mutants) and mAb. In brief, cell-free expressed P proteins were diluted and coated on microtiter plates at 4 °C overnight. After blocking with 1% bovine serum albumin, the two mAb (Genecreat, China) diluted in PBS were incubated with purified P domain proteins at 37 °C for 1 h. Following washing with PBST, HRP-conjugated goat anti-mouse IgG (Bioss, China) was added for incubating 30 min at 37 °C. After color development, read the absorbance at 450 nm. An OD_450_ of > twofold background after background subtraction was scored positive by the assay.

### Antibody blockade of P domain proteins binding assay

P proteins were pretreated with decreasing concentrations of mAbs for 1 h at 37 °C and added to wells coated with pig gastric mucin type III (PGM, Sigma-Aldrich, USA) and incubated for 1 h at 37 °C. Followed by washing steps, mouse anti-GII.17 P domain hyperimmune serum diluted 1:2000 in PBS was added and incubated for 1 h at 37 °C. After washing with PBST, HRP-conjugated goat anti-mouse IgG was added for incubating for 30 min at 37 °C. Blocking rates were calculated by comparing binding levels of in the presence and absence of mAbs. Sera from free P proteins-immunized animals were used as negative controls.

### Production of P proteins with mutations

Mutant P domain proteins with single or poly amino acid substitutions were designed and introduced into the GII.17 P domain using a wild-type templates. Site-directed residue mutations were performed using assembly PCR and the corresponding primer pairs (Additional File 2). After sequencing confirmation, mutant P domain proteins were expressed using the CFPS system and analyzed as described above.

### Structural modeling

Structural homology models representing the GII.17 capsid P domain were generated using the Swiss-Model server of the Swiss Institute of Bioinformatics [26], with default settings. Briefly, the Fasta format GII.17 capsid sequence was uploaded into the server, and the “Select Templates” option was selected. Models of P dimer was created using the templates and downloaded in.pdb format. Protein structures were visualized, and the figures were rendered using MacPyMOL version 2.1 (http://www.pymol.org). Selected of strains accession numbers are summarized in Additional File 3.

### Statistical analysis

Statistical analyses were performed using GraphPad Prism 7.04 (GraphPad Software, Inc, La Jolla, CA, USA). An analysis of variance (ANOVA) test was used to determine the mAb binding affinity between wild-type and mutant proteins. *P* value < 0.05 was considered significant difference.

## Results

### Genetic diversity and evolutionary analysis of GII.17

**Fig. 1:**
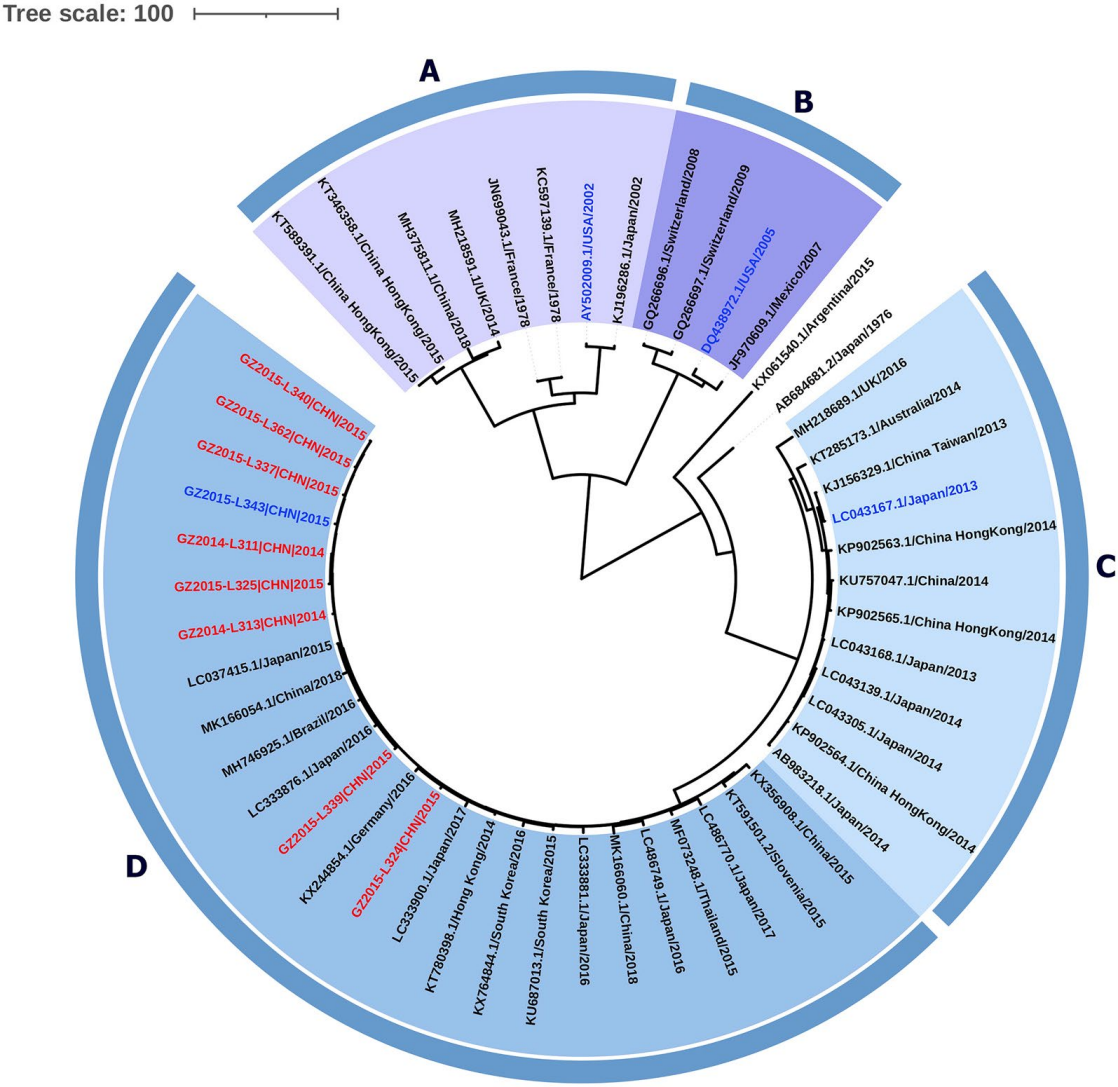
Human norovirus GII.17 phylogenetic tree. MCMC phylogenetic tree of GII.17 variants from 1978 to 2019 with indications of GenBank access codes, locations and isolation years. The tree was modified for visual performance: easy to distinguish different clusters. The eight strains were obtained from years of surveillance (red). The four selected variants were highlighted by blue represented each cluster

After removing redundant sequences from a total of 828 sequences (as of November 20, 2019), an analysis dataset consisting of 42 complete VP1 sequences was established. A further nine complete VP1 sequences obtained from our surveillance program were also included (highlighted in red in Fig. 1), resulting in a total of 51 complete VP1 sequences. The GII.17 genotype was divided into four clades (clades A-D), which mirrored previous studies [11]. Among these, strains in clade A covered the longest time period, ranging from 1978 to 2018 (Fig. 1, modified MCMC tree for visual performance; results of ML tree shown in Additional file 4: Figure S1 similar to MCMC tree). Most mutations were found in the P2 domain.

Four wild-type representative GII.17 variants were selected for comparison according to evolutionary analysis. The sequences identity of four strains were in the middle of respective cluster, therefore could better stand for each cluster. These variants were identified in 2002 (AY502009), 2005 (DQ438972), 2013 (LC043167), and 2015 (KT970376), each representing the four clades of the GII.17 genotype (clade A, B, C, and D, selected strains were highlighted in blue in Fig. 1). Based on amino acid alignment of the capsid, GII.17–2002, GII.17–2005, and GII.17–2013 exhibited 80.43%, 78.57%, and 93.10% identity, respectively, to GII.17–2015. These differences may contribute to antigenic and HBGA binding differences among the selected GII.17 variants.

### CFPS system-based synthesis of GII.17 P proteins

**Fig. 2:**
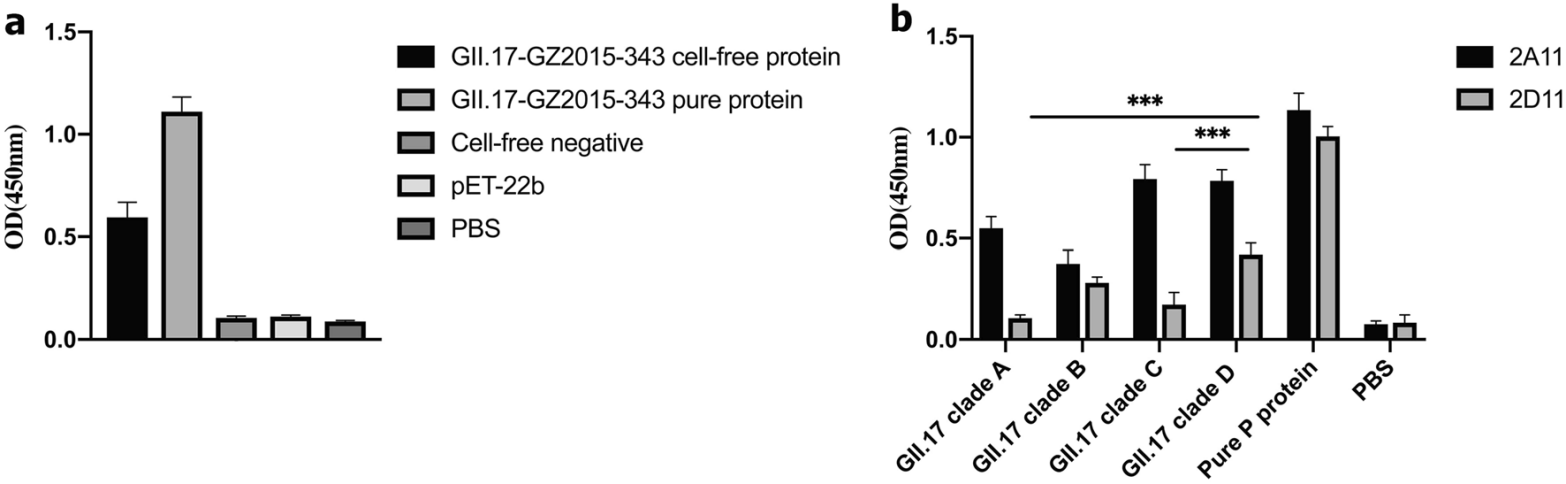
Confirmation of the validity of the CFPS system of producing norovirus P proteins. **a** Wild type GII.17 clade D variant was expressed by the CFPS system and was demonstrated by specific GII.17 antibody as well as its production yield compared to P proteins expressed by the cell-based system. **b** Four strains of GII.17 bound to two MAb. Only GII.17 clade B and GII.17 clade D were able to bind MAb 2D11, while all the four strains could interact with MAb 2A11. (*** *P* < 0.001)

To characterize whether cell-free proteins had antibody and ligand binding abilities, we performed an EIA assay for GII.17 clade D cell-free proteins, thereby determining their antibody binding abilities. As shown in Fig. 2a, compared with the positive and negative controls, GII.17 clade D cell-free proteins bound to mAb 2A11, as previously confirmed by *E. coli* BL21-expressed proteins. Four strains of GII.17 bound to mAb 2A11, whereas only GII.17 clade B and GII.17 clade D bound to mAb 2D11(GII.17–2005 and GII.17–2015, Fig. 2b). These EIA data indicated mAb 2A11 was with the broad reactivity pattern on GII.17 and the mAb 2D11 was more specific to recognize region on the GII.17 clade B and GII.17 clade D strain. Therefore, to investigate potential new antigenic features, we selected mAb 2D11 to screen a panel of mutant variants. To compare the relative binding of GII.17 clade D cell-free proteins to HBGAs, we examined these proteins for binding to PGM, a well-known norovirus ligand which covers the major known binding moieties of GII.17 strains [14, 27]. The results showed that the cell-free proteins bound to PGM with unstable results in the same dataset (Additional file 5: Figure S2).

Next, we analyzed protein production in the CFPS system and compared the mean optical density between the target proteins in the CFPS system and pure GII.17 clade D P proteins, finding that target protein production was at least 400 times higher than that in the cell-based *E. coli* system (based on binding ability).

### Residues 295–300 are key binding sites of the GII.17 antibody epitope

**Fig. 3:**
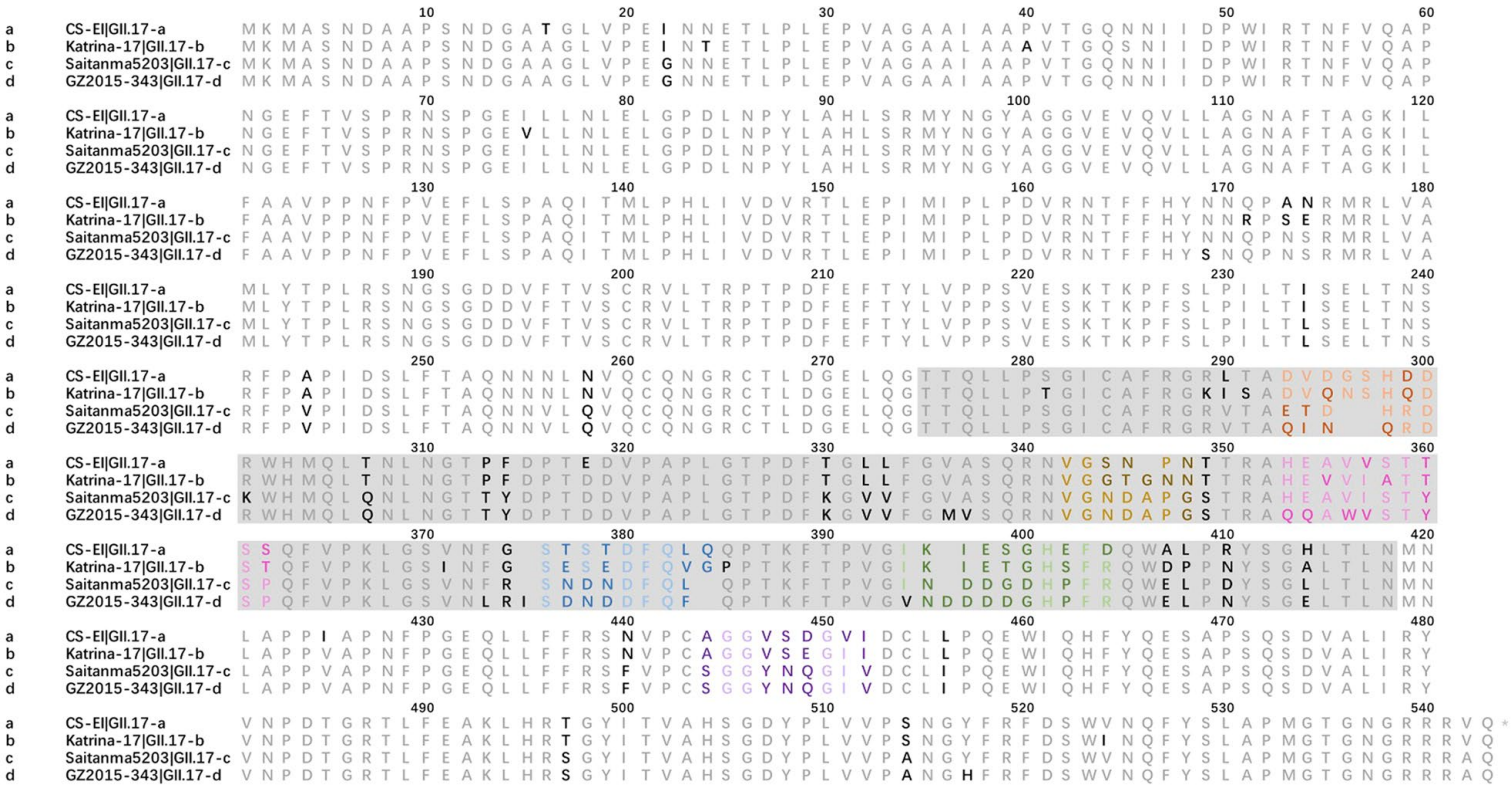
Amino acids alignment of selected variants. Residues that varied between the four variants were in boldface. Five loops known to interact with ligands and antibodies according to our previous bioinformatic analyses were highlighted with different colors (1oop 1: orange, loop 2: yellow, loop 3: blue, loop 4: dark green, loop 5: purple). P2 domain was shaded with grey

**Fig. 4:**
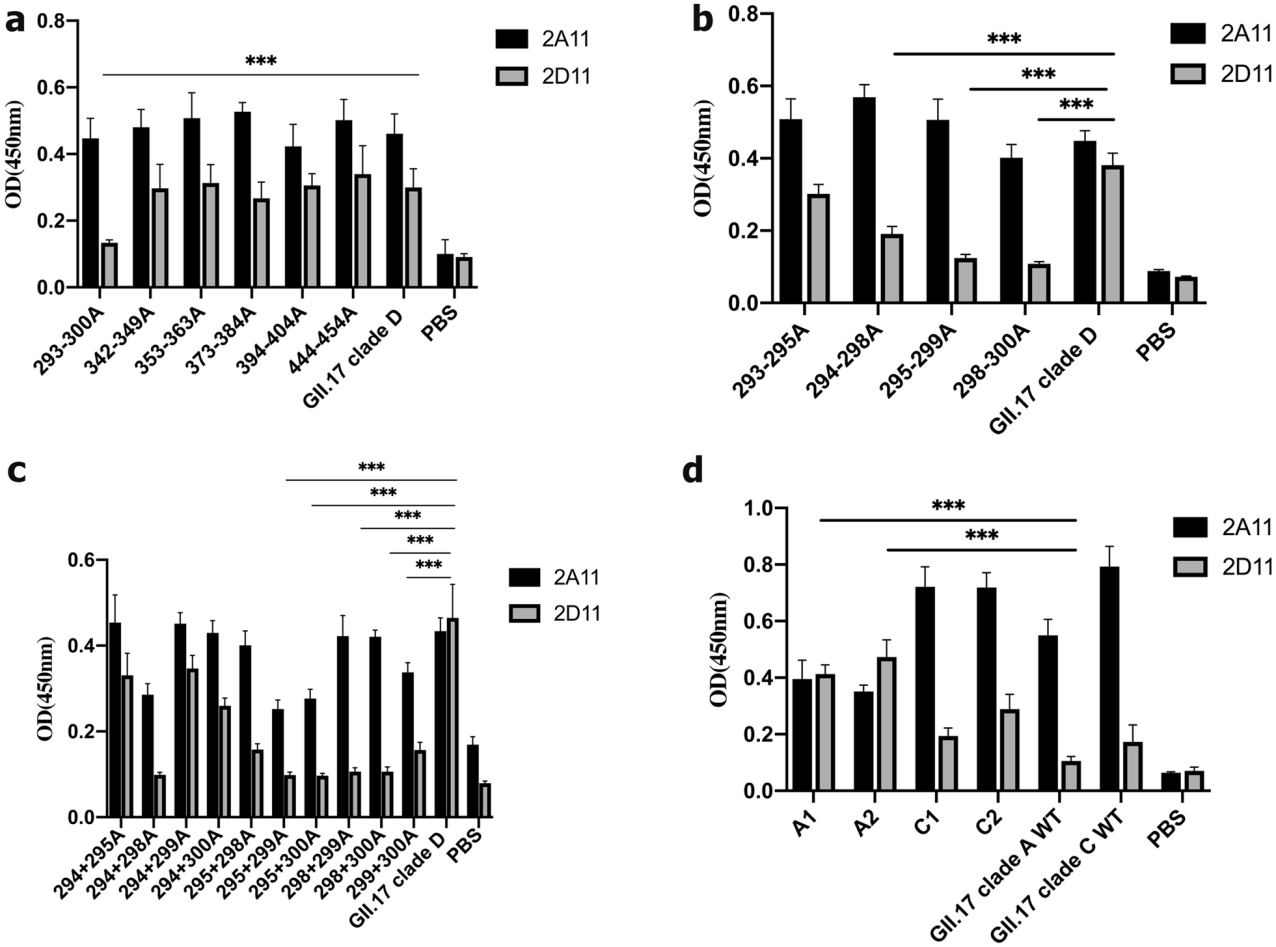
Residues of 295–300 were key binding sites for MAb 2D11. **a** To map the epitope for MAb 2D11, six variable regions were selected by an alignment of four time-ordered strains of GII.17 and sets of changes were introduced into these regions. Production of chimeric P proteins expressed by CFPS system was similar among the six mutants (evaluated by MAb 2A11) and residues 293–300 were found to lose binding affinity with MAb 2D11. **b** Continous three residues’ mutants were generated to narrow the binding site of MAb 2D11. Q293 was less relevant to MAb 2D11 binding. **c** Double alanine substitution mutants were introduced into 294-300aa. D300 existed across the four time-ordered strains of GII.17, but GII.17 clade A and GII.17 clade C could not restore the binding activity with MAb 2D11. Comparing these combined changing residues, loss of 298aa resulted in a decrease in binding of the antibody, while residues of 295, 299, and 300 might play a subordinate role in the binding site. The dotted line marked the negative control detection on panel B. **d** Binding of wild type P proteins of the GII.17 clade D variant, as well as reverse mutants to MAb 2A11 and 2D11. Residues of epitope A in GII.17 clade A and GII.17 clade C were replaced by those in GII.17 clade D. All reverse mutants restored the MAb 2D11 binding capability, while changes of these residues in GII.17 clade A resulted in the loss of binding capability with MAb 2A11. The statistical differences were shown by star symbols (*P < 0.05, ** *P* < 0.01, *** *P* < 0.001)

Genetic analyses indicated that the most variable amino acid changes among the four strains were in the P2 domain; as such, we introduced alanine to six potential epitopes in this domain. Reduced binding ability to mAb 2D11 indicated a unique sequence in residues 293–300, previously characterized as loop 1 (Fig. 3 and Fig. 4a) in GII.4 [28]. To examine the effects of this unique region, we created a panel of recombinant vectors with single site-directed residue mutations by replacing each residue in the region with alanine. Next, mutant variants were expressed using the CFPS system and characterized for mAb 2D11 binding using EIA (Additional file 6: Figure S3). The results indicated that no single mutation reduced the antibody binding capability, demonstrating that one residue was insufficient to affect antigenic properties. Next, four triple alanine substitution mutations were developed to dissect the role of residues 293–300. Although GII.17 clade D-293-295A restored mAb 2D11 binding, the remaining three mutant variants did not (Fig. 4b), indicating that residue 293 was less likely to affect mAb 2D11 binding. We continued to narrow the potential mAb 2D11-binding epitope. Ten additional double alanine substitution mutants were introduced to confirm the antibody-binding pocket. Considering the same residue in D300, we compared the relative binding activity of double alanine substitution mutants, finding that residue 298 was the most important factor affecting mAb 2D11 affinity. In addition, amino acids 295, 299, and 300 played a role in binding pocket formation (Fig. 4c). To further confirm the impact of the residues mentioned above, we created chimeric strains of GII.17 clade A and GII.17 clade C by substituting key residues in epitope A, previously identified in GII.17 clade D. The reason why we ignored GII.17 clade B was that GII.17 clade B and GII.17 clade D had similar binding properties with mAb 2D11. These data showed that amino acids 295–300 corresponded to the recognition site binding potency of mAb 2D11, while amino acids 293–294 promoted the binding capability (Fig. 4d).

### Structure of the GII.17 mAb 2D11-binding pocket

**Fig. 5:**
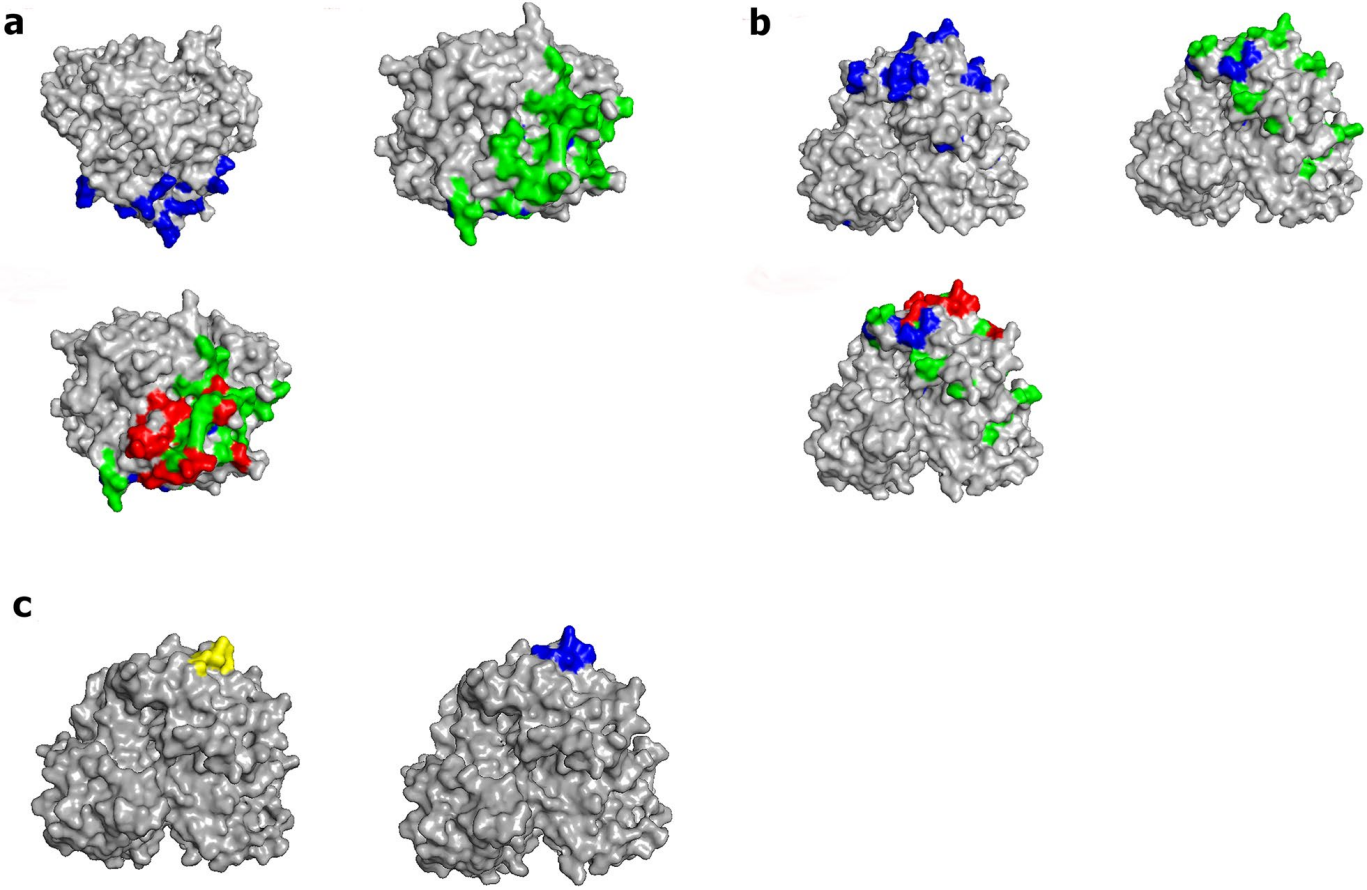
Map variable changes of GII.17 strains over time. **a** Side view of structure model of GII.17 clade A P domain dimer was shaded of grey. Residues changes between GII.17 clade A and GII.17 clade B (blue), between GII.17 clade B and GII.17 clade C (green), and between GII.17 clade C and GII.17 clade D (red). **b** Top views of the same model with (A). **c** Surface-exposed charge changes of residues of 293–300 in GII.17 clade D with alanine substitution (left side). Comparing with the wild type P domain structure model (right side), 293-300aa mutation in GII.17 clade D was likely to drove antigenic drift, losing the protrude motif

For visual purposes, homology models were displayed to highlight changes in residues between the GII.17 strains, as well as the conformational differences between mutants and wild types. When changing residues 293–300 to alanine in GII.17 clade D, mAb 2D11 binding was lost, confirming that these are the key residues of the antigenic epitope. Homology modeling of these residues showed that they formed a conformational epitope for the structural and functional integrity of the GII.17 variant, proximal to evolving epitope A. In the model, N295, Q298, R299, and D300 presented the P domain’s unique antigenic features, likely rearranging the local protrusion of epitope A (Fig. 5).

### PGM binding and blockade assay

**Fig. 6:**
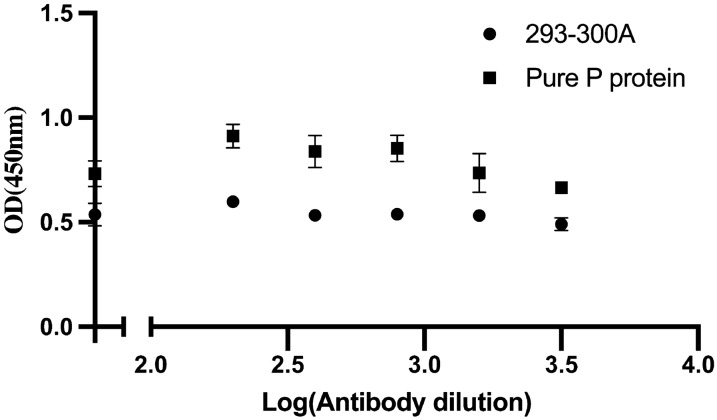
Ligand binding and blockade assay of GII.17 clade D-293-300A and wild type P protein expressed by cell-based system. Both of mutant 293-300A and wild type GII.17 clade D P protein bound pig gastric mucin III. Although different concentrations of antibody were used, the mutant retained the ligand-binding capability, which indicated that the epitope was irrelevant to receptor attachment

Although we demonstrated that mAb 2D11 recognizes a unique peptide, the ligand-binding assay failed to illustrate it using cell-free proteins. Self-assemble of dimers and particles may be restricted by platform components. Additionally, a previous study investigating CFPS system development for norovirus VLPs synthesis did not apply a binding assay [29], potentially explaining the same unstable results. To determine whether changes at residues 293–300 might be relevant to the blockade epitope, we expressed the pure protein of residue 293–300 substitution using the *E. coli* expression system, characterizing the protein using EIA and measuring blockade potency. The cell-based protein displayed similar mAb 2D11 binding activity to that of the proteins produced by the CFPS system. However, targeted changes in epitope A did not affect the binding potency of GII.17 clade D. Hence, these changed residues may only associate with antigenic drift (Fig. 6).

## Discussion

Genome analyses are a powerful tool to demonstrate the molecular evolution of the VP1 regions of GII.17 since its emergence. A GII.17-based MCMC time-scaled evolutionary phylogenetic tree showed that all the selected strains grouped into four clusters (clades A to D) [30, 31]. Apart from capsid gene, polymerase regions differences also contributed to the epidemiologic characteristics of the novel GII.17 strain [12, 32]. However, we did not assess the polymerase region of GII.17 because it was beyond our scope. Although predictive tools and empirical evidence continue to explore the dominant GII.4 genotypes, another strain GII.17 causing outbreaks should not be neglected. Unfortunately, limited information has been revealed regarding the cause of GII.17 outbreaks in the winter season of 2014–2015. To better understand the reason for the emergence of epidemic GII.17 clade D, we selected four representative strains, one from each cluster, to determine the effects of sequence change, thereby attempting to identify GII.17 antibody epitopes via P domain proteins.

To the best of our knowledge, this study is the first to report the antigenic epitope of GII.17 using rapid screening method, also providing direct evidence of the evolutionary mechanism in the major capsid gene of noroviruses. The sudden emergence of epidemic GII.17 strains emphasizes the need for understanding the norovirus molecular profile; thereby obtaining a global picture for vaccine development and molecular evolution. Novel GII.17 strains were expected to gain new antigenic features and a wider HBGAs binding profile compared to the previous strains of GII.17, which has been reported to result in the emergence of GII.17 [13, 14], allowing these variants to continually interact with new target populations [8].

Based on bioinformatics analyses and the conserved epitopes demonstrated in GII genogroup noroviruses, we previously deduced several potential epitopes of the new GII.17 variant [33]. However, few studies have reported any relevant evidence. To address these possibilities, we performed mutational analysis. Antibody responses were similar to the P proteins expressed in *E. coli* BL21, indicating that the cell-free system had translated the amino acids precisely. In this study, changes in residues 293–300 negatively affected mAb 2D11 binding, supporting their role in ligand binding and antigenicity. To confirm the key residues affecting antibody binding-related conformations, we then constructed a panel of mutants with alanine substitutions in amino acids 293–300. Substitutions of residues 295–300 resulted in a loss of mAb 2D11-binding activity. Furthermore, amino acid 298 appeared to be the key mAb 2D11-binding residue, while amino acids 295, 299, and 300 remained subordinate; but, the roles of subordinate residues should not be ignored. GII.17 clade A and GII.17 clade C strains with substitutions of residues as in GII.17 clade D confirmed this result. Together, these data provide valuable information for developing a vaccine with a possible neutralizing epitope.

Interestingly, two amino acids (I294 and N295) in epitope A did not interact with mAb 2D11 directly, rather mediating mAb 2A11 binding by changing particle conformation. Six continuous residues in the P domain, from amino acids 293 to 300, did not affect binding activity with mAb 2A11. Data of mAb 2A11 binding with reverse mutants GII.17 clade A mirrored this result. For GII.17 clade A, P protein bound to mAb 2A11 but could not react with mAb 2D11. It meant that P protein of GII.17 clade A could be expressed correctly without the specific epitope recognized by mAb 2D11. However, chimeric GII.17 clade A obtained the ability to bind to mAb 2D11 with lower protein yield (less binding to mAb 2A11), demonstrating mAb 2D11 recognized the residues of 293–300 on GII.17 clade D. Then we wondered whether 293-300aa on GII.17 clade D was involved in a ligand binding pocket. Although the data of binding assay did show the positive results, we could not obtain consistent results over three replicate experiments. Self-assemble may be restricted by platform components. Additionally, a previous study investigating CFPS system development for norovirus VLPs synthesis did not apply a binding assay [29], potentially explaining the same unstable results. To determine whether these corresponding changes associated with ligand recognition, we expressed mutant P domain proteins in a cell-based system. Results of the ligand-binding assay showed that substituted residues 293–300 could bind with PGM, indicating that these charge changes did not correlate well with ligand attachment.

Our site-directed mutagenesis analysis of GII.17 mirrored the findings of a recent report [34]. We and others’ results both showed that 293-300aa were an important epitope in GII.17 capsid gene, although different techniques were applied. The comparison reflected the advantages of the CFPS system: we used plasmids to perform mutagenesis analysis, while Yi et al*.,* might spend more time to express VLPs for screening mutants [34]. The putative antibody recognition site on the P proteins was similar to epitope A of GII.4 [35, 36]. Changes in GII.4 epitope A may have coincided with the appearance of a pandemic strain [37]. Furthermore, this epitope is also the main target for blocking antibodies detected in human polyclonal sera [38]. Several binding patterns were identified in GII.4 epitope A, including conformational restriction and linear sequence dependent [38], and the latter pattern matched our results for GII.17. Eight residues, with four binding sites, are present in epitope A of GII.4, namely, linear 294–298, 368, 372–373, and a rare group [39, 40]. Among these positions, we confirmed the linear epitope of residues 295–300, which is in line with GII.4. It has been reported that epitope A is hypervariable, with key residues loss correlating with viral escape from herd immunity [35]. The GII.17 clade C cluster, as opposed to previous GII.17 clade B and GII.17 clade D strains, could not bind mAb 2D11, confirming the existence of evolving epitopes, namely, those that escaped antibody recognition from mutations in and around surface-exposed regions that varied over time; epitope reemergence revealed evidence as to why the GII.17 pandemic emerged.

After decades of GII.4 pandemics, we learned that such epochal evolution in response to herd immunity leads to severe public health threats. Based on these data, we proposed a model in which several variable regions in the GII.17 sequence are fixed, with mutations in these regions likely restricted to several amino acids; this model was developed in GII.4, but without solid evidence [40]. Over time, norovirus strains have rapidly evolved, continuously escaping herd immunity. Understanding the specific residues and how noroviruses use them to evolve would greatly contribute to vaccine development. Ideally, a vaccine should contain all possible changes within the epitopes identified. Furthermore, unlike the 2014 epidemic, only a few cases of GII.17 norovirus infections have been reported in the past few years, suggesting that it evolved more rapidly than we thought [41]. As such, to develop a universal vaccine that covers both existing and future pre-pandemic norovirus strains, it is imperative that the proposed model be confirmed.

Owing to a lack of optimal cell culture system, various purified norovirus VLPs are in progress to better understand high-resolution molecular information related to evolution or vaccine research. Structural characterization of non-infected norovirus VLPs in complex with ligands or receptors is vital for providing a global picture of epitopes [42]. However, efforts to produce norovirus VLPs in insect cells are further impeded by the complex procedures and low yields of the correct architecture [29]. As such, mutational analysis of P proteins is well-accepted for mapping norovirus epitopes using cell-based produce systems, such as *E. coli* BL21. Considering this time-consuming procedure, it may be more appropriate to first apply the CFPS system to screen a panel of dozens of mutant proteins. Although self-assemble in the CFPS system was limited and protein expressed using the CFPS system could not bind to ligands, confirming the impact of key residues screened by the CFPS system via functional proteins using cell-based production systems could save much time and labor.

Limitations existed in this study. We did not use GII.17-specific mAbs that could map strain-specific motifs. As such, we could not investigate the effects of other epitopes on virus binding and neutralization. Although protein expressed using the CFPS system could not be calibrated accurately, the inability to absolute quantification could be compensated by relative quantitation. Another limitation is that the P proteins expressed using fast-expressing system is inability of binding with HBGAs. Further solutions to promote ligands binding stably with cell-free proteins are warranted.

## Conclusion

In conclusion, we demonstrated that the mAb 2D11-selected peptide matched several residues in the P domain, from amino acids 295 to 300, among which residue 298 played the most important role. mAb 2D11-recognized positions coincided closely with those recently identified as epitope A. Furthermore, our data indicated that the peptide matched a GII.17 linear anchor and confirmed that the CFPS system is a promising future tool for screening norovirus P protein mutants using mutational analysis.

## Supplementary Information

**Additional file 1:** Strains accession numbers used in phylogenetic trees construction.

**Additional file 2: Table S2.** Primers in this study.

**Additional file 3:** Strains accession numbers used in protein structural modelling.

**Additional file 4: Figure S1.** Human norovirus GII.17 phylogenetic tree using Maximum likelihood. The phylogenetic tree of GII.17 variants from 1978 to 2019 with indications of GenBank access codes.

**Additional file 5: Figure S2.** Cell-free P proteins function measured by ligand binding potency. Unlike P proteins expressed in cell-based system, unstable results of cell-free P proteins were shown in the continuous three times of independent cell-free expression

**Additional file 6: Figure S3.** Assessment of single mutants in residues of 293-300 binding to MAb 2D11. Production of chimeric P proteins of single mutation in residues 293-300 expressed by CFPS system was similar among the six mutants (evaluated by MAb 2A11). There was no difference in terms of the binding affinity with MAb 2D11 between these six mutants and wild type.

## Data Availability

Not applicable.
