# Supplementary material for: Rapid screening for antigenic characterization of GII.17 norovirus strains with variations in capsid gene
Source: Gut Pathog. 2022 Jul 25;14:31. doi: 10.1186/s13099-022-00504-1 (PMC9309444; doi:10.1186/s13099-022-00504-1)
Supplement: Supplementary file 2 — Additional file 2: Table S2. Primers in this study. [file 13099_2022_504_MOESM2_ESM.docx]

Supplementary Table 2. Primers in this study.

| Name | Primer sequence (5'-3') | Sense | Mutation |
| --- | --- | --- | --- |
| GII.17-A P1 | GGGAATTCCATATGTCTAAGACTAAACCCTTTTCTCT | - | Wild Type |
| GII.17-A P2 | CCGCTCGAGCTGCACCCGTCTGCGCCCA | + | Wild Type |
| GII.17-B P1 | GGGAATTCCATATG TCTAAGACAAAGCCTTT | - | Wild Type |
| GII.17-B P2 | CCGCTCGAGCTGCACTCTCCTGCGCCCATT | + | Wild Type |
| GII.17-C P1 | GGGAATTCCATATGTCTAAGACTAAGCCTTTT | - | Wild Type |
| GII.17-C P2 | CCGCTCGAGCTGAGCCCTCCTTCGCCC | + | Wild Type |
| GII.17-D P1 | GGGAATTCCATATGTCTAAAACTAAGCCTTTT | - | Wild Type |
| GII.17-D P2 | CCGCTCGAGGCAAAAGCAATCGCCACGGCAATCGCACTGAGCCCTCCTTCGCCCATT | + | Wild Type |
| P001 | GGCAGCTGCGGCAGCTGCTGCTGTCACCCGTC | - | 293-300A |
| P002 | GACGGGTGACAGCAGCAGCTGCCGCAGCTGCCAGGTGGCACATGCAACTGCAA | + | 293-300A |
| P003 | TGCGGCAGCCGCAGCAGCAGCCGCATTTCTTTGGCTTACCAT | - | 342-349A |
| P004 | TAAGCCAAAGAAATGCGGCTGCTGCTGCGGCTGCCGCAACCAGAGCCCAACAGGCGTGGGTT | + | 342-349A |
| P005 | TGCGGCCGCGGCGGCAGCAGCGGCAGCAGCGGCGCCCACTGGTGTGAATTTT | - | 394-404A |
| P006 | ACCAGTGGGCGCCGCTGCTGCCGCTGCTGCCGCCGCGGCCGCACAATGGGAACTACCAAACTAT | + | 394-404A |
| P007 | AGAACCTAATTTGGGGACAAATTGGGCGGCAGCGGCTGCAGCCGCCGCCGCTGCGGCTCTGGTTGA | - | 353-363A |
| P008 | TCAACCAGAGCCGCAGCGGCGGCGGCTGCAGCCGCTGCCGCCCAATTTGTCCCCAAATTAGGTTCT | + | 353-363A |
| P009 | TAGGTTCTGTCAATGCTGCTGCTGCTGCCGCTGCCGCCGCAGCAGCAGCACCGACAAAATTCACACCAGT | - | 373-384A |
| P010 | TAGGTTCTGTCAATTTTGGTAGTACTAGCACTGACTTCCAACTACAACAACCGACAAAAT | + | 373-384A |
| P011 | ATCCATTCTTGGGGAATGAGACAAGCTGCAGCAGCTGCGGCGGCAGCTGCTGCGCATGGCACGAAA | - | 444-452A |
| P012 | TTTCGTGCCATGCGCAGCAGCTGCCGCCGCAGCTGCTGCAGCTTGTCTCATTCCCCAAGAATGGAT | + | 444-452A |
| P013 | TGTCACGTTGGTTAATTGCTGCTGTCACC | - | Q293A |
| P014 | GGTGACAGCAGCAATTAACCAACGTGAC A | + | Q293A |

Supplementary Table 1. (Continued)

| Name | Primer sequence (5'-3') | Sense | Mutation | |  |  |
| --- | --- | --- | --- | --- | --- | --- |
| P015 | CCACCTGTCACGTTGGTTAGCTTGTGCTGT | - | I294A | |  |  |
| P016 | ACAGCACAAGCTAACCAACGTGACAGGTGG | + | I294A | |  |  |
| P017 | TGCCACCTGTCACGTTGGGCAATTTGTGCTGT | - | N295A | |  |  |
| P018 | ACAGCACAAATTGCCCAACGTGACAGGTGGCA | + | N295A | |  |  |
| P019 | ATGTGCCACCTGTCACGTGCGTTAATTTG | - | Q298A | |  |  |
| P020 | CAAATTAACGCACGTGACAGGTGGCACAT | + | Q298A | |  |  |
| P021 | GCATGTGCCACCTGTCAGCTTGGTTAATTTG | - | R299A | |  |  |
| P022 | CAAATTAACCAAGCTGACAGGTGGCACATGC | + | R299A | |  |  |
| P023 | AGTTGCATGTGCCACCTGGCACGTTGGTTAAT | - | D300A | |  |  |
| P024 | ATTAACCAACGTGCCAGGTGGCACATGCAACT | + | D300A | |  |  |
| P025 | GTGCCACCTGTCACGTTGGGCAGCTGCTGCTGTCACC | - | 293-295A | |  |  |
| P026 | GGTGACAGCAGCAGCTGCCCAACGTGACAGGTGGCAC | + | 293-295A | |  |  |
| P027 | CATGTGCCACCTGTCACGTGCGGCAGCTTGTGCTGT | - | 294-298A | |  |  |
| P028 | ACAGCACAAGCTGCCGCACGTGACAGGTGGCACATG | + | 294-298A | |  |  |
| P029 | TTGCATGTGCCACCTGTCAGCTGCGGCAATTTGTGCTGTC | - | 295-299A | |  |  |
| P030 | GACAGCACAAATTGCCGCAGCTGACAGGTGGCACATGCAA | + | 295-299A | |  |  |
| P031 | AGTTGCATGTGCCACCTGGCAGCTGCGTTAATTTGTGC | - | 298-300A | |  |  |
| P032 | GCACAAATTAACGCAGCTGCCAGGTGGCACATGCAACT | + | 298-300A | |  |  |
| P033 | TGCCACCTGTCACGTTGGGCAGCTTGTGCTGTC | - | 294+295A | |  |  |
| P034 | GACAGCACAAGCTGCCCAACGTGACAGGTGGCA | + | 294+295A | |  |  |
| P035 | ATGTGCCACCTGTCACGTGCGGCAATTTGTGCT | - | 295+298A | |  |  |
| P036 | AGCACAAATTGCCGCACGTGACAGGTGGCACAT | + | 295+298A | |  |  |
| P037 | AGTTGCATGTGCCACCTGTCAGCTGCGTTAATTTGTGC | | | - | | 298+299A |

Supplementary Table 1. (Continued)

| Name | Primer sequence (5'-3') | Sense | Mutation |
| --- | --- | --- | --- |
| P038 | GCACAAATTAACGCAGCTGACAGGTGGCACATGCAACT | + | 298+299A |
| P039 | AGTTGCATGTGCCACCTGGCAGCTTGGTTAATTTG | - | 299+300A |
| P040 | CAAATTAACCAAGCTGCCAGGTGGCACATGCAACT | + | 299+300A |
| P041 | TTGCATGTGCCACCTGTCACGTGCGTTAGCTTGTGCTGTC | - | 294+298A |
| P042 | GACAGCACAAGCTAACGCACGTGACAGGTGGCACATGCAA | + | 294+298A |
| P043 | TTGCATGTGCCACCTGTCAGCTTGGGCAATTTGTGCTGTC | - | 295+299A |
| P044 | GACAGCACAAATTGCCCAAGCTGACAGGTGGCACATGCAA | + | 295+299A |
| P045 | TTGCATGTGCCACCTGGCACGTGCGTTAATTTGTGC | - | 298+300A |
| P046 | GCACAAATTAACGCACGTGCCAGGTGGCACATGCAA | + | 298+300A |
| P047 | TTGCATGTGCCACCTGTCAGCTTGGTTAGCTTGTGCTGTCAC | - | 294+299A |
| P048 | GTGACAGCACAAGCTAACCAAGCTGAC AGGTGGCACATGCAA | + | 294+299A |
| P049 | TTGCATGTGCCACCTGGCACGTTGGGCAATTTGTGCTGT | - | 295+300A |
| P050 | ACAGCACAAATTGCCCAACGTGCC AGGTGGCACATGCAA | + | 295+300A |
| P051 | TTGCATGTGCCACCTGGCACGTTGGTTAGCTTGTGCTGTCAC | - | 294+300A |
| P052 | GTGACAGCACAAGCTAACCAACGTGCCAGGTGGCACATGCAA | + | 294+300A |
| P053 | TGCATGTGCCATCTGTCACGTTGGGCTGCGTTTACATCTGCTT | - | D295-300a |
| P054 | AAGCAGATGTAAACGCAGCCCAACGTGACAGATGGCACATGCA | + | D295-300a |
| P055 | AACTGCATGTGCCATCTGTCACGTTGGCTTCCGTTAATTTGTGCTGTTAGTC | - | D293-300a |
| P056 | GACTAACAGCACAAATTAACGGAAGCCAACGTGACAGATGGCACATGCAGTT | + | D293-300a |
| P057 | AGTTGCATGTGCCACTTGTCACGTTGGTTAGTTTCTGCTGTC | - | D295-300c |
| P058 | GACAGCAGAAACTAACCAACGTGACAAGTGGCACATGCAACT | + | D295-300c |
| P059 | AGTTGCATGTGCCACTTGTCACGTTGGTTAATTTGTGCTGTCACC | - | D293-300c |
| P060 | GGTGACAGCACAAATTAACCAACGTGACAAGTGGCACATGCAACT | + | D293-300c |
