# Supplementary material for: Rapid screening for antigenic characterization of GII.17 norovirus strains with variations in capsid gene
Source: Gut Pathog. 2022 Jul 25;14:31. doi: 10.1186/s13099-022-00504-1 (PMC9309444; doi:10.1186/s13099-022-00504-1)
Supplement: Supplementary file 3 — Additional file 3: Strains accession numbers used in protein structural modelling. [file 13099_2022_504_MOESM3_ESM.docx]

Supplementary Table 3. Strains accession numbers.

| Variants | GeneBank Accession number | PDB ID for model building |
| --- | --- | --- |
| NoV-CS-EI\|GII.17-a | AY502009.1 | 5ZUQ |
| NoV-Katrina-17\|GII.17-b | DQ438972.1 | 5F4O |
| NoV-Saitanma5203\|GII.17-c | LC043167.1 | 5F4J |
| NoV-GZ2015-343\|GII.17-d | KT970376.1 | 5ZUS |
