# Supplementary figures and images for: Rapid screening for antigenic characterization of GII.17 norovirus strains with variations in capsid gene
Source: Gut Pathog. 2022 Jul 25;14:31. doi: 10.1186/s13099-022-00504-1 (PMC9309444; doi:10.1186/s13099-022-00504-1)

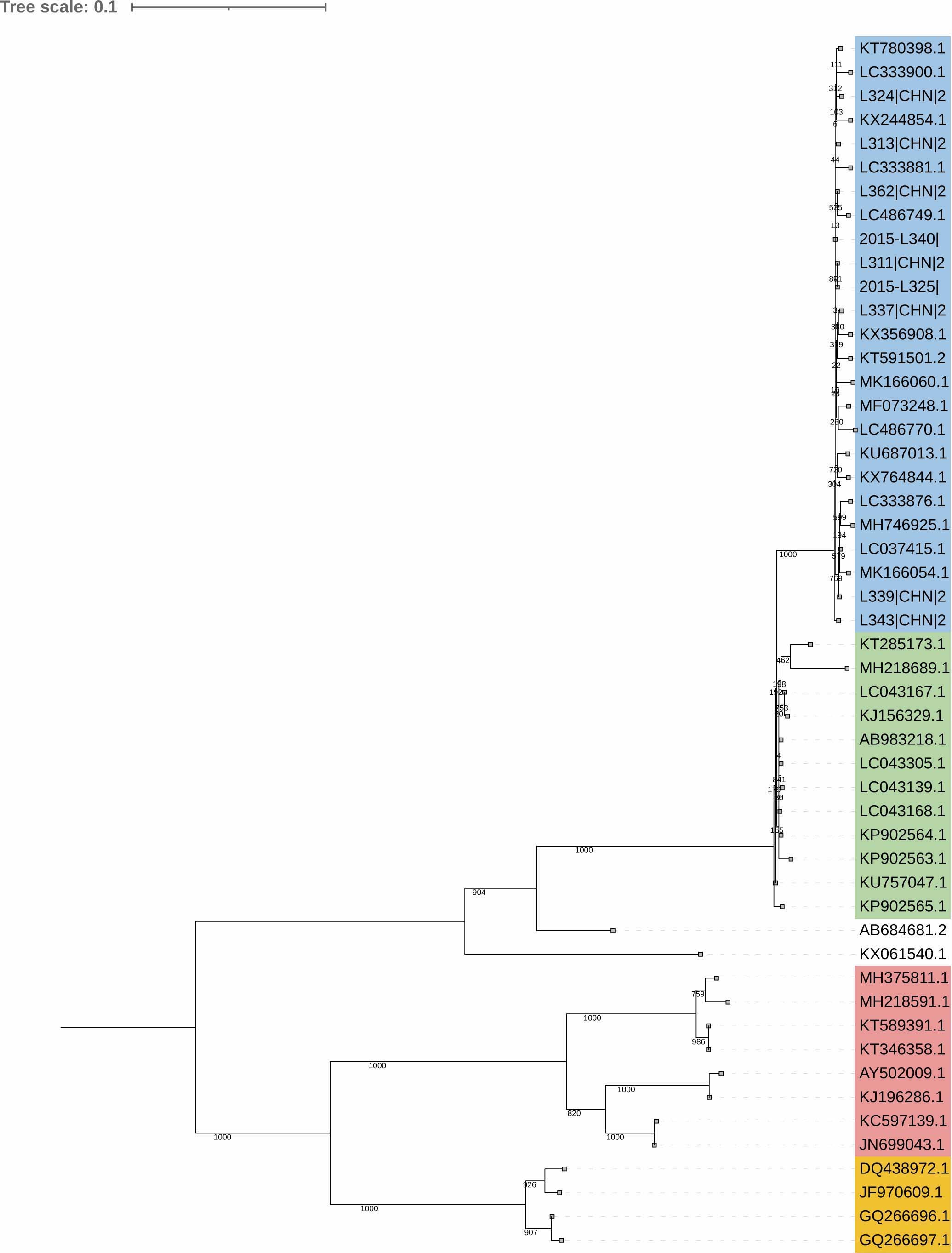

Supplement: Supplementary file 4 — Additional file 4: Figure S1. Human norovirus GII.17 phylogenetic tree using Maximum likelihood. The phylogenetic tree of GII.17 variants from 1978 to 2019 with indications of GenBank access codes. [file 13099_2022_504_MOESM4_ESM.jpg]

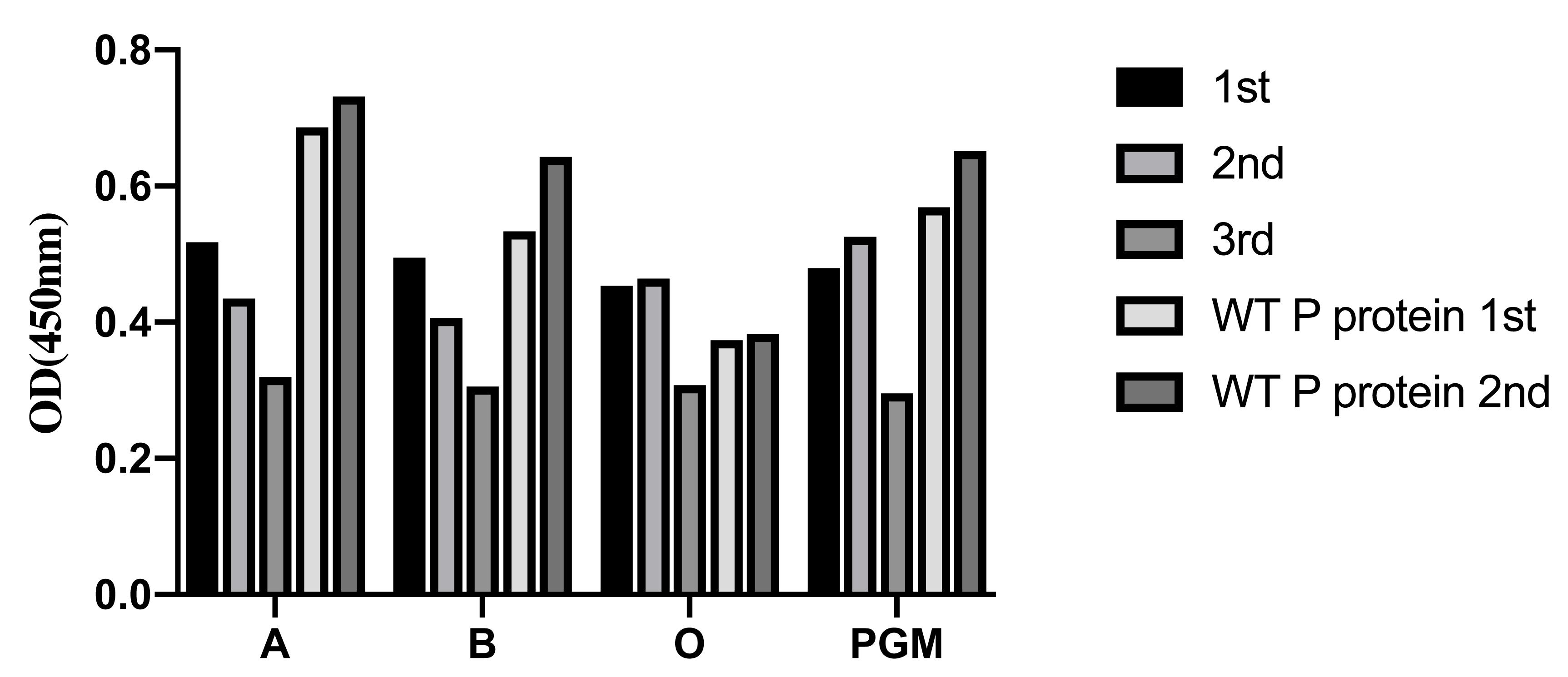

Supplement: Supplementary file 5 — Additional file 5: Figure S2. Cell-free P proteins function measured by ligand binding potency. Unlike P proteins expressed in cell-based system, unstable results of cell-free P proteins were shown in the continuous three times of independent cell-free expression [file 13099_2022_504_MOESM5_ESM.jpg]

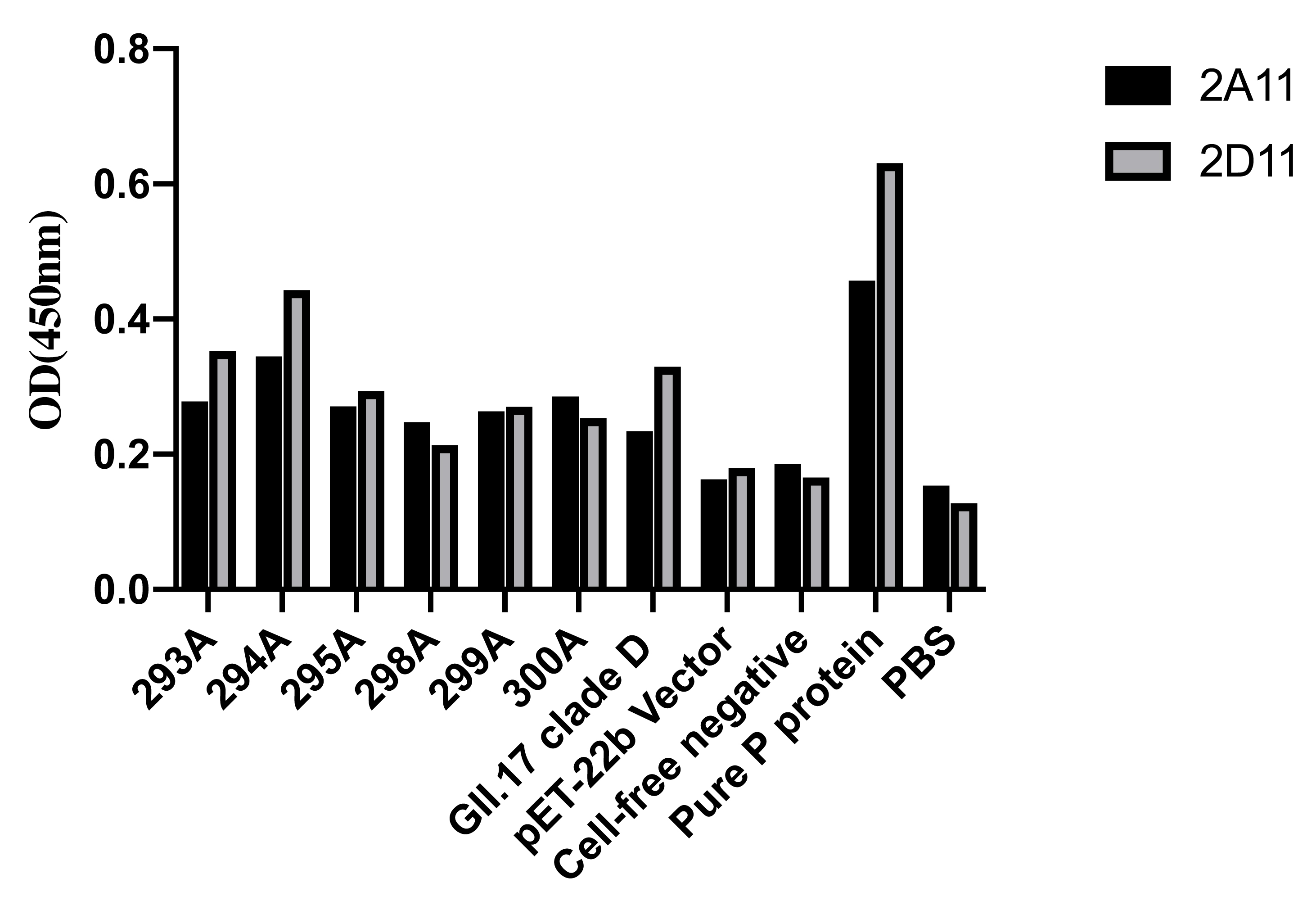

Supplement: Supplementary file 6 — Additional file 6: Figure S3. Assessment of single mutants in residues of 293-300 binding to MAb 2D11. Production of chimeric P proteins of single mutation in residues 293-300 expressed by CFPS system was similar among the six mutants (evaluated by MAb 2A11). There was no difference in terms of the binding affinity with MAb 2D11 between these six mutants and wild type. [file 13099_2022_504_MOESM6_ESM.jpg]
